# Amino Acid Supplementation Affects Imprinted Gene Transcription Patterns in Parthenogenetic Porcine Blastocysts

**DOI:** 10.1371/journal.pone.0106549

**Published:** 2014-09-02

**Authors:** Chi-Hun Park, Young-Hee Jeong, Yeun-Ik Jeong, Jeong-Woo Kwon, Taeyoung Shin, Sang-Hwan Hyun, Eui-Bae Jeung, Nam-Hyung Kim, Sang-Kyo Seo, Chang-Kyu Lee, Woo-Suk Hwang

**Affiliations:** 1 Sooam Biotech Research Foundation, Seoul, Republic of Korea; 2 Department of Animal Sciences, College of Veterinary Medicine, Chungbuk National University, Cheongju, Chungbuk, Republic of Korea; 3 College of Veterinary Medicine, College of Veterinary Medicine, Chungbuk National University, Cheongju, Chungbuk, Republic of Korea; 4 Animal Quarantine Division, Gyeonggi-Do, Suwon, Republic of Korea; 5 Department of Agricultural Biotechnology, Animal Biotechnology Major, and Research Institute for Agriculture and Life Science, Seoul National University, Seoul, Republic of Korea; USA, United States of America

## Abstract

To determine whether exogenous amino acids affect gene transcription patterns in parthenogenetic porcine embryos, we investigated the effects of amino acid mixtures in culture medium. Parthenogenetic embryos were cultured in PZM3 medium under four experimental conditions: 1) control (no amino acids except L-glutamine and taurine); 2) nonessential amino acids (NEAA); 3) essential amino acids (EAA); and 4) NEAA and EAA. The rate of development of embryos to the four-cell stage was not affected by treatment. However, fewer (*P*<0.05) embryos cultured with EAA (12.8%) reached the blastocyst stage as compared with the control group (25.6%) and NEAA group (30.3%). Based on these findings, we identified genes with altered expression in parthenogenetic embryos exposed to medium with or without EAAs. The results indicated that EAA influenced gene expression patterns, particularly those of imprinted genes (e.g., *H19*, *IGF2R*, *PEG1*, *XIST*). However, NEAAs did not affect impaired imprinted gene expressions induced by EAA. The results also showed that mechanistic target of rapamycin (*MTOR*) mRNA expression was significantly increased by EAA alone as compared with control cultures, and that the combined treatment with NEAA and EAA did not differ significantly from those of control cultures. Our results revealed that gene transcription levels in porcine embryos changed differentially depending on the presence of EAA or NEAA. However, the changes in the *H19* mRNA observed in the parthenogenetic blastocysts expression level was not related to the DNA methylation status in the *IGF2/H19* domain. The addition of exogenous amino acid mixtures affected not only early embryonic development, but also gene transcription levels, particularly those of imprinted genes. However, this study did not reveal how amino acids affect expression of imprinted genes under the culture conditions used. Further studies are thus required to fully evaluate how amino acids affect transcriptional regulation in porcine embryos.

## Introduction

Amino acids were originally considered to be energy sources, biosynthetic precursors, and physiological regulators. However, it is becoming increasingly clear that amino acids also participate in several crucial biological processes, such as the regulation of gene expression and as cell signaling molecules [1], [2].

Amino acids have been detected in the reproductive tract and shown to play essential roles in the development of preimplantation mammalian embryos [3], [4]. Preimplantation embryos can consume and produce amino acids, but the net rates of depletion or appearance vary among amino acids [5]. Moreover, amino acids exert stage-dependent effects on preimplantation embryonic development, with different amino acids required for different roles. [4]. Although amino acids are not required for complete preimplantation development of mouse embryos [6], some, but not all, of amino acid supplementation has been reported to improve the *in vitro* blastocyst development rate of mouse zygotes [7], [8]. There are 20 amino acids which are routinely supplemented in embryonic culture media of various species with the classical sets of essential (EAA) and non-essential (NEAA) amino acids, commercially available mixtures, based on the formulation originally constructed by Eagle in 1959 [9], although it has been suggested that all 20 Eagle's amino acids may not be essential for developing embryos (for a review, see Summers and Biggers. [10]). Numerous studies have shown that the presence of NEAA benefits the preimplantation development of embryos; whereas, EAA impair development during the cleavage stages [11], [12]. Alternatively, reducing EAA concentrations in culture media improves preimplantation development and the development of embryos to full-term viable offspring [13]–[15]. Despite increased efforts to determine the amino acid requirements for preimplantation development, the mechanisms of action of amino acids on embryonic development are not well defined. Previous studies using endpoint-based approaches have provided relatively limited information of the effects of amino acid supplementation on blastocyst formation rates, total cell number, and apoptotic incidence [14].

Suboptimal *in vitro* culture media, such as the addition of serum or deficiency of amino acids, leads to retarded development and aberrant gene expression in early-stage embryos [16], [17]. Several lines of evidence demonstrate that changes in imprinted methylation and expression can be induced under *in vitro* conditions, and that these alterations irreversibly influence further embryonic and placental development [18]–[21]. Nevertheless, the ways in which these factors affect methylation imprint marks and cause these perturbations in embryos during culture are poorly understood.

The ability of amino acids to control gene expression *via* activation of transcription factors in mammalian cells under conditions of excess or deficiency has been increasingly recognized [22]. Among amino acids, glutamine and arginine have been shown to modulate gene expression at the transcriptional and post-transcriptional levels [23]. A recent study demonstrated that arginine and leucine activate trophoblast motility in mouse embryos by modulating the mechanistic target of rapamycin (MTOR) pathway [24], [25]. Little attention has been given to the roles of amino acids in the control of embryonic gene expression. We thus hypothesized that gene expression patterns in early embryos could be affected by the inclusion of exogenous amino acids in culture media.

To investigate this hypothesis, we examined changes in gene transcription in blastocysts cultured in the presence of different sets of amino acids using the following four treatments: control (no amino acids except L-glutamine and taurine), NEAA, EAA, and NEAA + EAA. In the present study, we used parthenogenetic embryos because the current porcine IVF program presents challenges mainly due to the high incidence of polyspermy (proximately 30∼40%) and thus such afflicted embryos exhibit an imbalance in metabolism. The amino acid response patterns between polyspermic and normally fertilized embryos may vary [26]. In addition, a large number of autosomal genes as well as X-linked genes display sexual dimorphic transcription pattern in preimplantation embryos at the blastocyst stage [27]. In this regard, using parthenotes may be an adequate model for studying the transcriptional effects of amino acid supplements while making it possible to minimize these confounding variables that are present in the porcine IVF system.

Previous studies have found altered mRNA expression patterns in *in vitro* embryos as compared with those derived *in vivo*. *BEX1* (brain-expressed X-linked gene 1), *G6PD* (glucose-6-phosphate dehydrogenase), *H19* (H19 gene), *NNAT* (neuronatin), and *XIST* (X inactivation-specific transcript) were upregulated and *IGF2R* (insulin-like growth factor 2 receptor) and *PGK1* (phosphoglycerate kinase 1) were downregulated in *in vitro* embryos as compared with *in vivo* derived embryos [27]. *GRB10* (growth factor receptor-bound protein 10) and *PEG1* (paternally expressed gene 1) mRNA levels were not different between *in vivo* and *in vitro* embryos [28]. Thus, we considered the observed trends in up-regulation or down-regulation of mRNA expression to be largely due to the *in vitro* environment used during culture. In addition, six metabolism-related genes, *HKII* (hexokinase II), *ACACA* (acetyl-CoA carboxylase 1), *PCmt* (mitochondrial pyruvate carboxylase), *GLUT2* (glucose transporter 2), *MCT1* (monocarboxylate transporter 1), *SLC3A1* (solute carrier family 3), and *MTOR* were included in the study.

## Materials and Methods

### Ethics Statement

The pig experiments were carried out in strict accordance with the recommendations in the Guide for the Care and Use of Laboratory Animals of the National Veterinary and Quarantine Service and were supervised by Gyeonggido Livestock and Veterinary Service. Each study was approved by the animal ethics committee of Sooam biotech research foundation (license number AEC-20081021-0001). Porcine ovaries were provided by the regional slaughterhouse (Hyup-Shin, Anyang, Korea).

### *In vitro* maturation (IVM)

Porcine ovaries were transported to the laboratory in 0.9% (w/v) NaCl supplemented with 100 mg/ml streptomycin sulfate (Amresco, Solon Ind.) within 1 h of collection at 37°C. Cumulus-oocyte complexes (COCs) were obtained from follicles 3–6 mm in diameter using 18-gauge micro needles. Oocytes possessing an evenly granulated cytoplasm and a compact surrounding cumulus mass were collected, and washed twice with TL-Hepes-PVA medium (Tyrodes lactate-Hepes medium supplemented with 0.01% polyvinyl alcohol). After washing, 40–50 COCs were transferred to 500 µl of an IVM medium (TCM-199; Invitrogen, Carlsbad, CA) supplemented with 10 ng/ml epidermal growth factor (EGF), 1 µg/ml insulin (Sigma-Aldrich Corp.), 4 IU/ml eCG (Intervet, Boxmeer, The Netherlands), hCG (Intervet) and 10% (v/v) porcine follicular fluid (pFF) and were cultured for 22 h. After 22 h of culture, the COCs were transferred to an IVM medium without hormones and were cultured for a further 22 h at 38.5°C in an atmosphere containing 5% CO_2_ and 100% humidity.

### Parthenogenesis

Briefly, cumulus-free oocytes were activated by an electric pulse (1.0 kV/cm for 60 µsec) in activation medium (280 mM mannitol, 0.01 mM CaCl_2_, 0.05 mM MgCl_2_) using a BTX Electro-cell Manipulator (BTX, CA, USA), followed by 4 h of incubation in PZM3 medium containing 2 mmol/l 6-dimethylaminopurine.

### *In Vitro* Culture (IVC)

We cultured 50–80 post-activated oocytes in 4-well dishes containing 500 µl of PZM3 for 144 h. Embryo culture conditions were maintained at 38.5°C in an atmosphere containing 5% CO_2_, 5% O_2_ and 100% humidity. The EAA for Eagle basal medium without glutamine and NEAA for minimum essential medium were used to treat embryos, based on the findings of a previous study [29]. The concentration of each amino acid in PZM3 was as follows: alanine, 0.10; arginine, 0.10; aspartic acid, 0.10; cystine 0.05; glutamic acid, 0.10; glycine, 0.10; histidine, 0.04; isoleucine, 0.20; leucine, 0.20; lysine, 0.20; methionine, 0.05; phenylalanine, 0.10; proline, 0.10; serine, 0.10; threonine, 0.20; tryptophan, 0.02; tyrosine, 0.10; valine, 0.20 (mmol/g).

### Cell number counts

The zona pellucida was removed from embryos using Tyroid's acid solution (Gibco). The number of cells for blastocysts cultured at Day 6 was counted after staining with DAPI and under UV using a fluorescence microscope in three independent experiments.

### mRNA synthesis and qPCR

mRNA from single or pooled embryos was extracted using the Dynabeads mRNA Direct Kit (Dynal Asa, Oslo, Norway) according to the manufacturers' instruction. cDNA synthesis was performed with the High Capacity cDNA Reverse transcription kit (Applied Biosystems; ABI, Foster City, CA). Using a final volume of 20 µl the procedure was carried out at 37.5°C for 60 min, and samples were subsequently incubated at 95°C for 5 min to inactivate reverse transcriptase. The qPCR experiments were based on our previous studies [27], [28]. To minimize the effect of variability of individual sample quality, amplification yield for each sample was primarily analyzed using quantitative real-time PCR analysis with two housekeeping genes, *ACTB* and *RN18S*. Prior to use for the experiment, cDNA samples with proper melt-curve peak and threshold cycle (Ct) values were selected and frozen. Amplification and detection were carried out with the ABI 7300 Real-Time PCR system (ABI) using Power SYBR Green PCR master mix (ABI) under the following conditions: 95°C for 15 min, 40 cycles of denaturation at 95°C for 15 s and annealing at 60°C for 60 s. Each sample was run at least in duplicate. All the Ct values of tested genes were normalized to *ACTB* and *RN18S* expression, and relative expression ratios were calculated via the 2^−ΔΔ^ Ct method [30]. The efficiency of PCR amplification for all the primer pairs (Table 1) was calculated from a serial dilution of an adult fibroblast cDNA, and their specificity was confirmed via sequencing analysis.

**Table 1:** Primer sequences for qRT-PCR.

Gene	Primer sequence 5′-3′	Gene Access no.	Length (bp)
*HKII*	F:CCGAGCCCGCCAGAA	NM_001122987	90
	R:CGCTCCATTTCCACCTTCAT		
*ACACA*	F:CAGCATCTCCAACTTCCTTCACT	NM_001114269	102
	R:TTGACTCCCCCATAGATAAGTTCAA		
*PCmt*	F:CCACAACTTCAGCAAACTCTTCAG	NM_214349	90
	R:CGCCAAGGGCACTCATACA		
*GLUT2*	F:CCGCTGGATGACCGAAAA	NM_001097417	85
	R:TTGGATCCCCTGGGTATGGT		
*MCT1*	F:CATCGCCATCTCTCCAAAGTG	EU587017	90
	R:TCTTTGTCGCTGTCCTTGAAAG		
*SLC3A1*	F:ATTATTGGCGGCTGCTTGTC	NM_001128445	90
	R:AATGACTCCAATGCACAAGTAAAGTT		
*MTOR*	F:CGCCTGAACACGTGGTAATAGA	XM_003127584	116
	R:CAGGCATACGGTCGAGACTTAA		

The qPCR primers for *GRB10*, *H19*, *IGF2R*, *NNAT*, *PEG1*, *BEX1*, *G6PD*, *PGK1*, *XIST*, *ACTB*, and *RN18S* were from the published papers [27], [28].

### DNA isolation and bisulfite treatment

To estimate the methylation status of the *IGF2/H19* Differentially Methylated Region (DMR) 3, genomic DNA from pools of 50 parthenogenetic blastocysts was isolated. The isolation of genomic DNA from porcine samples was carried out using a commercial spin column (G-spin Genomic DNA extraction kit for Cell/Tissue, iNtRON, Korea). The genomic DNA was digested with EcoRI (New England Biolabs, Germany). The Bisulfite treatment of DNA was performed with the EZ DNA Methylation- GoldTM kit (Zymo Reserch, USA) according to the manufacturer's instructions. Nested PCR of bisulfite-treated DNA was carried out using the primer for DMR3 in *IGF2*/*H19* locus from the published paper [31]. The PCR amplification was performed with a 2× PCR master mix solution (iNtRON, Korea) containing 0.5 pmol of the primers. The first-round of PCR was performed as follows, 1 cycle of 94°C for 10 min; 35 cycles of 95°C for 45 sec/50°C for 1 min/72°C for 1 min, 72°C for 7 min. The nested PCR was carried out at 1 cycle of 94°C for 10 min; 40 cycles of 95°C for 45sec/55°C for 2 min/72°C for 2 min; 1 cycle of 72°C for 7 min. PCR products were cloned into the pGEMT-Easy vector (Promega) and transformed into E. coli cells (Novagen, USA) and at least 10 insert positive plasmid clones were sequenced using an ABI PRISM 3730 automated sequencer (Applied Biosystems). The methylation patterns were analyzed in sequences derived from clones with ≥ 98% cytosine conversions only. All experiments were repeated at least three times for DMR3. The methylation level in each sample was determined by dividing the number of methylated CpG sites by the total number of CpG sites in ten or more sequenced clones.

### Statistical Analysis

The data obtained in this study were analyzed using the GraphPad Prism statistical program (GraphPad Software, San Diego, CA). Data on developmental rates and gene expression data were arcsine transformed and then examined using analysis of variance (ANOVA) and Dunnett's test. Relative transcription levels the means of two groups were analyzed using Student's t tests. All data are expressed as mean values ± SEM. A probability of *P*<0.05 was considered statistically significant.

## Results

### Effect of essential and non-essential amino acids on embryo development

**Figure 1:**
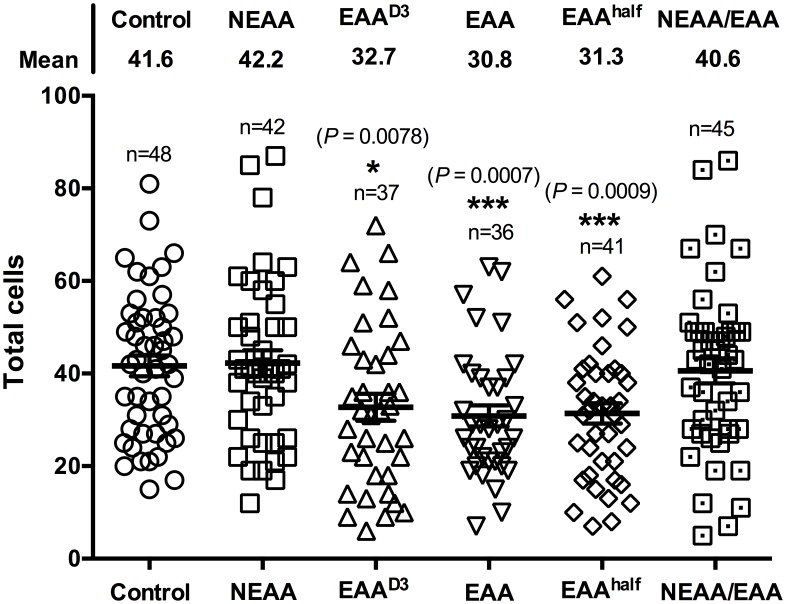
Total cell numbers of parthenogenetic blastocysts cultured with NEAA or EAA or both. Scatter dot plots represent average numbers of total cells in control (41.6; *n* = 48), NEAA (42.2; *n* = 48), EAA^D3^ (32.7 *n* = 37), EAA^D6^ (30.8; *n* = 36), EAA^half^ (31.3; *n* = 41), and NEAA + EAA (40.5; *n* = 45) blastocysts at Day 6. Cells were counted under UV using a fluorescence microscope with a DAPI fluorescence emission filter in three independent experiments. *P*-values are derived when treatments are compared to control (*, *P*<0.05, **, *P*<0.001).

We initially compared the effects of the inclusion of EAA and/or NEAA in culture media on preimplantation development of electrically activated parthenogenetic zygotes. Based on a previous finding [29], EAA and NEAA were added to PZM3 culture medium in concentrations within the physiologically normal ranges for the entire culture period. We did not consider the effects of L-glutamine and taurine because they are basic components of culture media. For each set of experiments, a total of 1,718 oocytes were divided into four groups of 30–50 embryos each and were activated by electrical stimulation to form parthenotes. After 48 h of culture, the cleavage rates of embryos were similar among groups (64.4–77.1%; Table 2) In contrast, fewer (*P*<0.05) embryos cultured with EAA (12.8%) reached the blastocyst stage as compared with all other groups (NEAA, 30.3%; NEAAs + EAA, 28.2%; control, 25.6%). However, rates of blastocyst formation by embryos treated with NEAA and NEAA + EAA did not differ from the control group. Figure 1 shows the total numbers of cells in the blastocysts at the end of the culture period (144 h). Embryos were randomly selected for cell count analysis regardless of quality. Like the developmental rate, the cell number was reduced (*P*<0.05) in blastocysts cultured with EAA (32.7) as compared with all other groups (NEAA, 40.4; NEAA + EAA, 40.5; control, 41.6). However, this analysis also revealed that the presence of EAA in two groups increased the number of poor-quality blastocysts with few cell numbers (<20) as compared with values for blastocysts in the control and NEAA treatment groups. We also compared cell numbers in blastocysts cultured with EAAs with those cultured using only PZM3 at a later developmental stage (48–144 h) and at half-strength concentration. We found that the dilution of EAA to one-half the original concentration (EAA^half^) and the delay of addition of EAA until 48 h after initiation of culture (EAA^D3^) had no significant effect on rate of development as compared with the original EAA treatment after 144 h (data not shown). Furthermore, cell numbers did not differ significantly among the EAA treatments (Figure 1). These results indicate that the presence of EAA alone during culture impaired development of porcine embryos, but that this adverse effect was diminished when EAA were administered with NEAA. These results indicate the extent to which the inclusion of amino acids in culture media might be beneficial in late-stage embryonic development.

**Table 2:** Effects of amino acids on the development of parthenogenetic embryos.†

NEAA	EAA	No. examined	No (%). cleaved		No (%). blastocyst	
(−)	(−)	517	373	(71.7±4.0)	129	(25.6±4.2)
(−)	(+)	520	402	(77.1±3.4)	75	(12.8±4.0)*
(+)	(−)	340	220	(64.4±2.9)	99	(30.3±4.5)
(+)	(+)	341	238	(68.9±2.7)	94	(28.2±3.7)

† The number of replicates was 10. The cleavage and blastocyst rates were recorded 48 h and 144 h post-activation, respectively. All percentage data expressed show mean values ± SEM. The asterisk indicates a significant difference determined by Analysis of Variance (*P*<0.05).

### Changes in gene transcription in embryos cultured with amino acids

**Figure 2:**
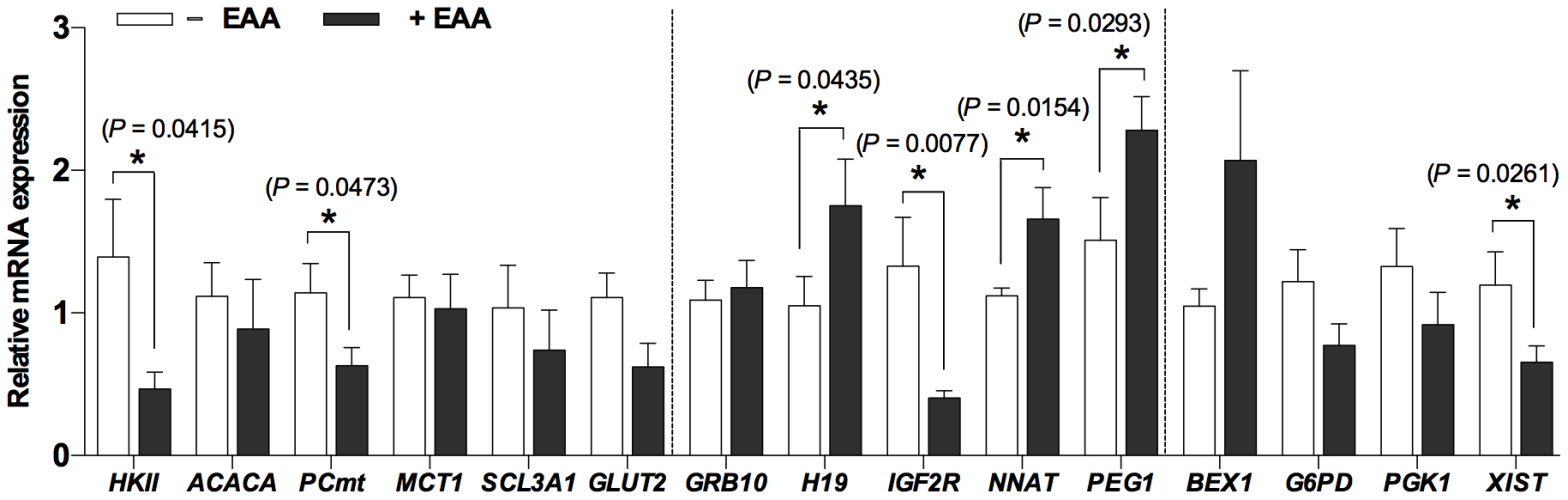
Real-time PCR analysis of mRNA expression levels in parthenogenetic blastocysts cultured with/without EAAs. The relative mRNA expression of the selected 15 genes; *HKII*, *ACACA*, *PCmt*, *MCT1*, *SLC3A1*, *GLUT2*, *GRB10*, *H19*, *IGF2R*, *NNAT*, *PEG1*, *XIST*, *BEX1*, *G6PD*, and *PGK1*, in EAA blastocysts (*n* = 30) after normalization relative to the average of *RN18S* and *ACTB* (internal control) gene, were compared to those of the blastocysts cultured in the absence of amino acid mixtures (control) which was set to 1. Bars with asterisks indicate significantly different from the value in the control (*, *P*<0.05). Data is mean ± S.E.M.

To identify altered mRNA expression in the presence of EAA in culture media, quantitative real-time polymerase chain reaction assays were carried out to measure expression of 15 genes related to metabolism (*HKII, ACACA*, *PCmt*, *GLUT2*, *MCT1*, and *SLC3A1*) and imprinting (*GRB10*, *H19*, *IGF2R*, *NNAT*, and *PEG1*), as well as X-linked genes (*XIST*, *BEX1*, *G6PD*, and *PGK1*). For this comparison, we used a total of 3 cDNA samples generated from pooled blastocysts (*n* = 5) from each group. The levels of expression of mRNAs differed significantly in the presence of EAA for seven genes (Figure 2). EAAs significantly increased expression of *H19*, *NNAT*, and *PEG1* mRNAs, but reduced expression of *HKII*, *PCmt*, *IGF2R*, and *XIST* mRNAs as compared with controls (*P*<0.05). There was a trend for reduced expression of *BEX1* (*P* = 0.0643). The observed differences may be caused by factors other than amino acids, including a high degree of transcriptional variation among embryos in each group.

**Figure 3:**
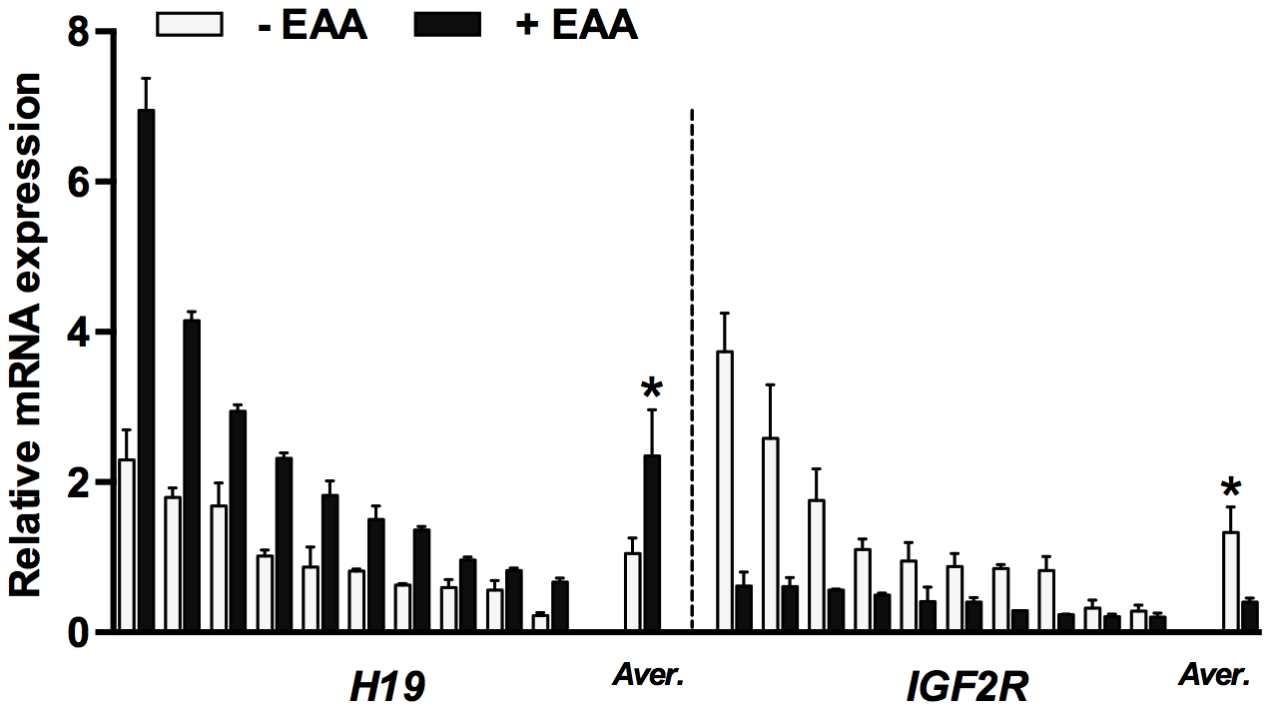
mRNA expression pattern of *H19* and *IGF2R* in individual parthenogenetic blastocysts. The quantitative data represents the values from transcripts of *H19* and *IGF2R* genes in individual mRNA samples (*n* = 20). Bars with asterisks indicate significantly different from the value in the control (*, *P*<0.05). Data is mean ± S.E.M.

To confirm gene expression differences observed in the pooled embryo cDNA samples, *H19* and *IGF2R* transcript levels were analyzed in individual blastocysts (*n* = 10 for each groups) are shown in Figure 3. In the presence of EAA, *H19* expression increased, but was more variable among individual embryos; conversely, the level and variability of *IGF2R* expression decreased. As seen in the developmental rates, mRNA-level responses to EAA^half^ and EAA^D3^ found little or no difference in gene transcription levels between low-concentration and high-concentration treatments. The inclusion of EAA in culture media at any stage of embryonic development induced the same change in mean expression levels (data not shown).

**Figure 4:**
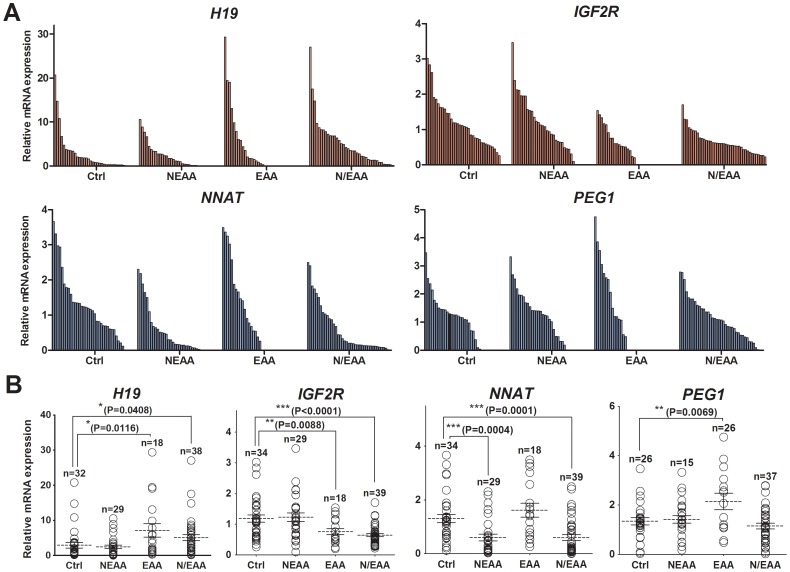
Differential mRNA expression patterns for four imprinted genes in parthenogenetic blastocysts cultured with NEAA or EAA or both. Bars (A) and aligned dot plots (B) represent mRNA transcript levels of the imprinted *H19*, *IGF2R*, *NNAT* and *PEG1* genes in individual blastocysts after normalization relative to the average of *RN18S* and *ACTB* (internal control) genes, were compared to those of control blastocysts cultured in the absence of amino acid mixture, which was set to 1. The aligned dot plots with asterisks indicate significantly different from 1 (*, *P*<0.05; **, *P*<0.001; ***, *P*<0.0001).

Next, we explored whether EAA-induced changes in the expression of identified imprinted genes could be compensated for by the addition of NEAA, as seen for developmental competency (Table 2 and Figure 1). For comparison, a total of 119 individual cDNA samples were isolated from individual blastocysts (control, *n* = 31; NEAA, *n* = 29; EAA, *n* = 20; NEAA + EAA, *n* = 39). Figure 4 shows distinct transcriptional patterns for four imprinted genes in response to the amino acid treatments. *H19* mRNA expression increased in response to EAA (*P* = 0.01) and NEAA + EAA (*P* = 0.04) as compared with the control and NEAA-only treatments. Similarly, *IGF2R* expression decreased in response to EAA (*P* = 0.01) and NEAA + EAA (*P*<0.0001) as compared with the other treatment group. In contrast, *NNAT* expression decreased in response to NEAA (*P* = 0.0001) and NEAA + EAA (*P* = 0.0004) as compared with the control and EAA-only groups. In the case of *PEG1*, mRNA levels increased (*P* = 0.0069) in response to EAA as compared with the control group. The results show that the four imprinted genes exhibited distinct patterns of expression in blastocysts cultured with the three amino acid treatments.

**Figure 5:**
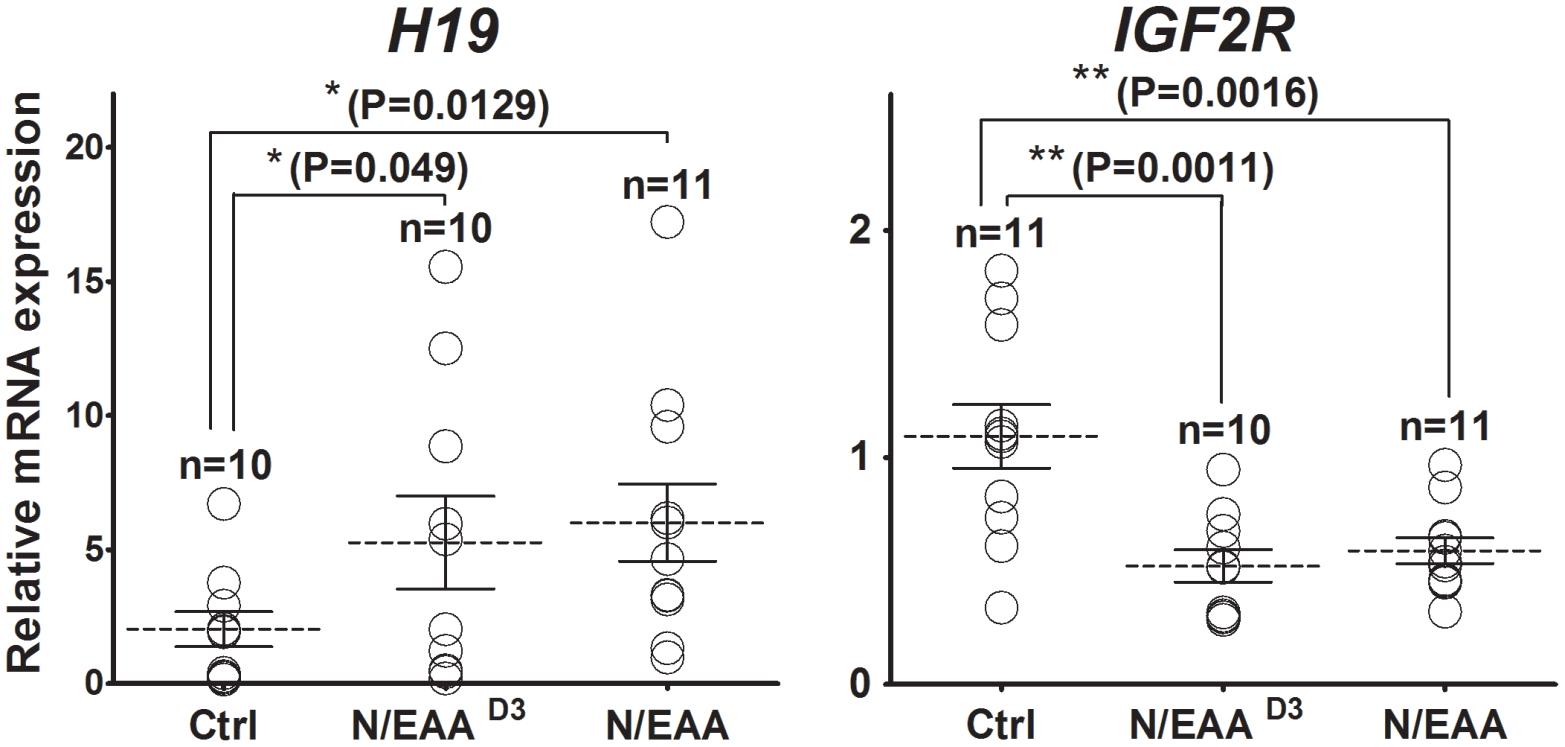
*H19* and *IGF2R* mRNA levels between the amino acid treatments after 48 h of culture and for whole embryo culture. The embryos (NEAA/EAA^D3^) were cultured with NEAAs for the first 48 h. Then, these embryos were transferred and subsequently cultured in PZM3 supplemented with NEAA + EAA up to 144 h. The quantitative data represents the values from transcripts of *H19*: A and *IGF2R*: B genes in individual mRNA samples (*, *P*<0.05; **, *P*<0.001).

In addition, we estimated mRNA levels of *H19* and *IGF2R* in response to treatment after 48 h of culture (NEAA/EAA^D3^; Figure 5) to find that gene expression levels did not differ from those of whole embryos after 144 h of culture.

### DNA methylation patterns in *IGF2/H19* DMR 3 in parthenogenetic blastocysts

**Figure 6:**
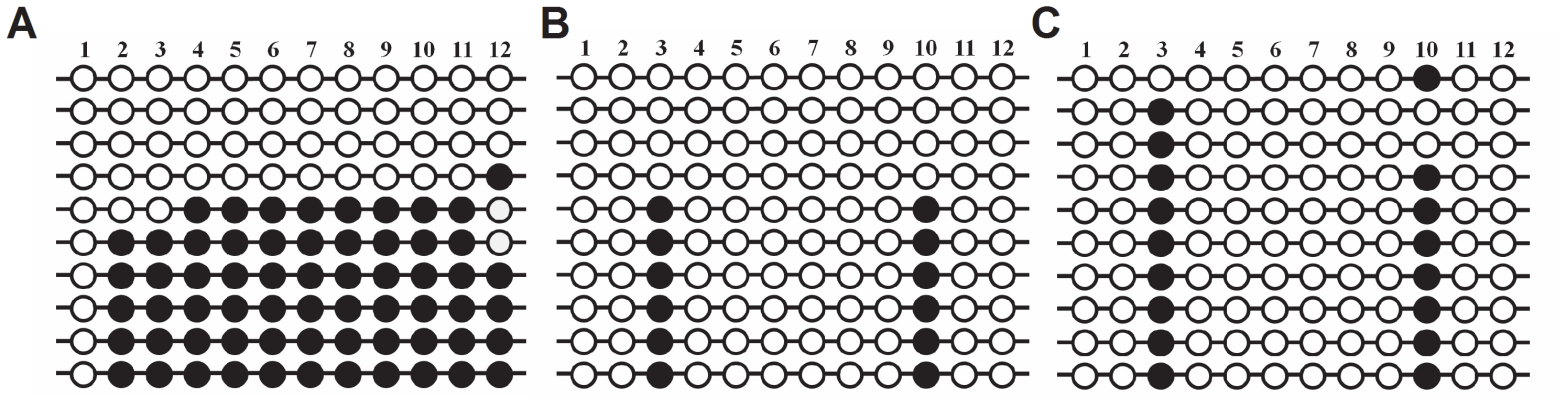
Methylation status of *IGF2/H19* DMR3 in porcine parthenogenetic blastocysts. The methylation patterns of DMR3 in porcine: A, adult liver tissue; B, control parthenogenetic blastocysts (*n* = 50); and C, blastocysts cultured with EAA (*n* = 50). Individual circles indicate a CpG dinucleotide. Open and solid circles represent unmethylated and methylated CpGs, respectively.

We sought to determine if the differential transcriptional patterns of imprinted genes in blastocysts cultured in the presence of EAA were affected by changes in DNA methylation. The methylation status of *IGF2/H19* DMR 3 in parthenogenetic blastocysts was investigated using a bisulfite genomic sequencing assay [31]. The results showed that these regions were hemimethylated in adult liver tissue, with an average methylation level of 41% (Figure 6A–C). Regardless of EAA treatment, most CpGs in parthenogenetic blastocysts had nearly identical methylation patterns and were typically hypomethylated with reference to their uniparental origin. These results indicate that the changes in imprinted mRNA expression observed in the parthenogenetic blastocysts due to the inclusion of EAAs may not be related to the degree of DNA methylation in this region.

### Effect of amino acids on *MTOR* mRNA expressions

**Figure 7:**
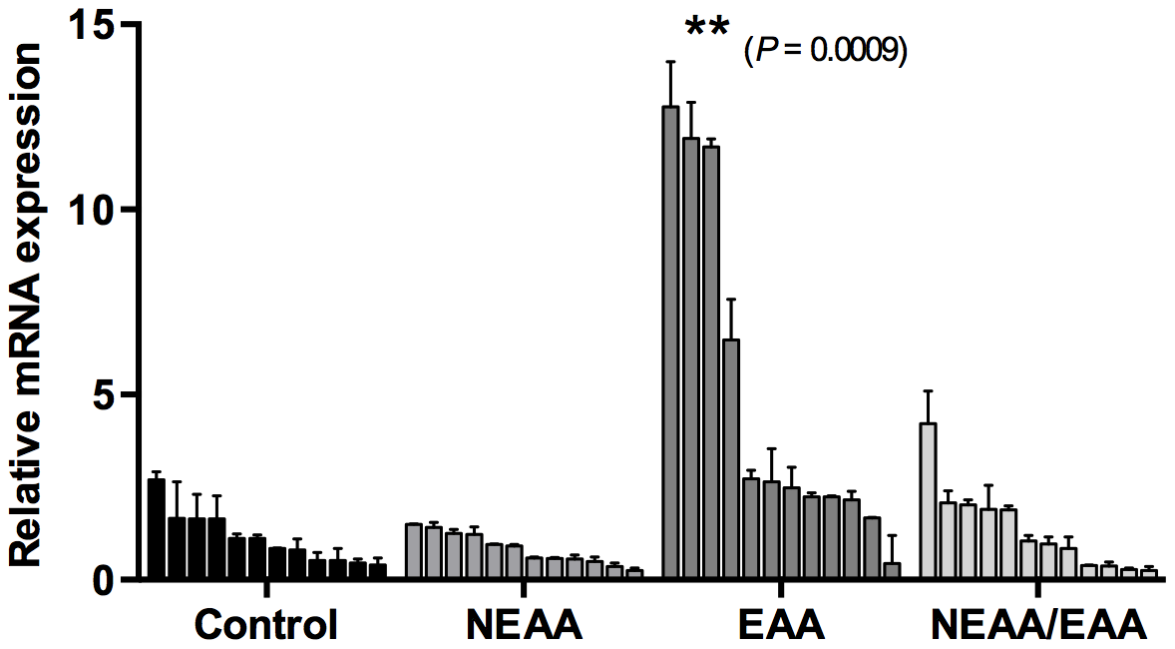
*MTOR* mRNA expression in parthenogenetic blastocysts cultured with NEAA or EAA or both. The values from transcripts of *MTOR* gene in individual samples of each group (*n* = 12) are expressed as mean ± S.E.M. The means of each AA treatment group were compared to those of control blastocysts. Bars with asterisks indicate significant difference between in control and amino acid treatment groups (**, *P*<0.001).

MTOR has a pivotal role in the activation of trophectoderm outgrowth, which is regulated by specific amino acids, such as arginine and leucine [32]. Thus, we further investigated the effects of different amino acid treatments on expression of *MTOR* mRNA. *MTOR* mRNA levels differed (*P* = 0.0009) between the EAA-supplemented and control groups (Figure 7). However, transcript abundance did not differ between the NEAA + EAA and control groups. These results indicated that treatment with EAA alone significantly increased *MTOR* gene expression, but that this action may be inhibited by combined treatment with NEAA.

## Discussion

Although not all amino acids appear to be equally effective in stimulating early embryonic development, amino-acid supplementation of culture media improves development of embryos to the blastocyst stage [8]. Emerging evidence suggests that amino acids regulate gene expression by activating transcription factors or regulatory sequences that respond to certain amino acids, such as glutamine and arginine [2]. Here, we determined that gene transcription patterns in parthenogenetic porcine embryos are altered by the inclusion of amino acids in media. The four imprinted genes tested displayed distinct transcriptional patterns in response to EAA or NEAA. However, no link may exist between the *H19* mRNA expression level and the DNA methylation status in the *IGF2/H19* domain.

The present study showed decreased rates of blastocyst formation and blastocyst cell numbers in parthenogenetic embryos cultured with EAA. The addition of NEAA compensated partially for the detrimental impacts of EAA on preimplantation embryonic development. These findings support previous observations in several species [12], [33]. Surprisingly, the adverse effects were not alleviated by changes in EAA concentration or length of exposure, and NEAA + EAA treatment was not stimulatory, as reported by others [13], [15]. Also, cleavage-stage embryos cultured with EAA or NEAA + EAA displayed increases in poor-quality blastocysts containing a small number of nuclei, albeit at a lower rate. Despite reduced blastocyst formation, EAA in the medium did not influence development to 4-cells after culture for 2 days. The findings of the current study are inconsistent with those of Van Thuan et al. [12], who found that development of parthenogenetic porcine zygotes was severely arrested by treatment with EAA in medium for the first 48 h of culture. It has been suggested that this effect was caused mainly by the coordinated presence of some nonpolar EAA, such as valine, leucine, isoleucine, and methionine [12]. The discrepancy between these results and ours may be explained by the use of different culture medium. The previous study used Whitten's medium with no amino acids included which perturbs expression of imprinted genes in mouse embryos [18]. PZM3 medium containing serum albumin and two amino acids (L-glutamine and taurine) was used in this study. Thus, comparison of the results of the two studies must be undertaken with caution. Taken together, these results indicate that the addition of amino acids to embryo culture medium has little or no additive effect on preimplantation development of parthenogenetic porcine embryos in terms of blastocyst formation rate and numbers of cells per blastocyst. However, whether the adverse effect of EAA is caused by certain nonpolar EAA remains unclear. Further studies taking these issues into account are required.

The present study showed that the expression of glycolysis-related genes (*HKII* and *PCmt*) was significantly reduced in response to EAA. Amino acids play a pivotal role in the metabolic and homeostatic regulation of preimplantation embryos, and *in vitro* embryos have the ability to adapt to their environment [34]. HK is the first rate limiting glycolytic enzyme, plays a key regulatory role in glucose metabolism. The previous study demonstrates that the glucose-deprived media can downregulate the expression of glycolytic genes in yeast [35]. Thus, these results may indicate that increased amino acid availability improved their ability to meet metabolic requirements in response to the absence of appropriate nutrients, such as glucose [14].

The results of this study also revealed that the expression of several imprinted genes was affected by the inclusion of amino acid mixtures in the culture medium. Expression of *H19* increased while expression of *IGF2R* decreased in response to EAAs; whereas, *NNAT* expression decreased in response to NEAA. In addition, *PEG1* expression increased in blastocysts with the addition of EAA alone, and this enhanced expression appears to have been compensated for by the inclusion of NEAA. The overexpression of *H19* in mouse embryos induces lethality after embryonic day 14 [36], and the loss of *Igf2r* imprinting causes fetal and placental overgrowth in mice [37]. In pig embryos, expression of *H19* and *NNAT* mRNAs is elevated and *IGF2R* expression is reduced in *in vitro* derived as compared with *in vivo* derived embryos [28]. These results indicate that EAA have a negative effect on *H19*, *IGF2R*, and *PEG1* mRNA expression, whereas NEAA do not affect expression of those genes. In contrast, NEAA reduced *NNAT* mRNA levels, but EAA did not affect expression. These results indicate that the addition of NEAA alone may be beneficial for appropriate *NNAT* mRNA expression in preimplantation porcine embryos under the employed culture conditions. Also, the addition of NEAA appears to offset the deregulated expression of the *PEG1* gene induced by EAA. This was not seen with *H19* and *IGF2R*. Taken together, these findings suggest that such supplementation could affect gene transcription levels in parthenogenetic porcine embryos during culture, particularly EAA alters expression of the *H19* and *IGF2R* genes.

Imprinted genes control the placental supply of nutrients for fetal growth *via* epigenetic mechanisms, including DNA methylation under the control of environmental factors and nutrients [38]. The DNA methylation status in the *Igf2/H19* imprinted region is fundamental for allele-specific imprinted *H19* expression [39]. Doherty et al. [18] demonstrated that disrupted *H19* expression observed in Whitten's medium with no amino acid supplementation was associated with reduced methylation levels, while the embryos cultured in KSOM supplemented with amino acids exhibited proper imprinted gene expression. Similar results have been found in other studies of mouse embryos cultured in KSOM medium [21], [40]. However, no evidence for an alteration in DNA methylation at the *IGF2/H19* locus in parthenogenetic porcine embryos was found in this study. We previously reported that no altered methylation pattern, but the increased *H19* mRNA expression, was found in biparental porcine blastocysts produced by *in vitro* fertilization [28]. Thus, the deregulation of *H19* mRNA expression in porcine embryos may not be associated with DNA methylation status at this locus. It is likely therefore that *in-vitro* culture induced epigenetic alterations do not necessarily entail similar mechanisms between different species. The exact cause is unknown, but it might be related to other mechanisms that regulate its expression, such as p53 protein [41]. Taken together, these results suggest that the current *in vitro* culture medium used in this study stably maintained DNA methylation in the imprinted *H19* domain in preimplantation porcine embryos. Caution must be applied in interpreting these isolated findings, which may not be transferable to the overall epigenetic stability of other imprinted loci or developmentally important genes. Although this study did not evaluate development or expression under conditions deprived of all amino acids, the medium may have contributed to the disruption of proper DNA methylation in embryos.

The results of this study show that *MTOR* mRNA levels were significantly increased by the addition of EAA (including arginine and leucine). This finding further supports previous research, which found that MTOR signaling stimulates proliferation and migration of trophectoderm cells and is regulated by specific amino acids, such as arginine and leucine [31], [42]. A very recent study demonstrated that arginine, leucine, and glutamine stimulate proliferation of trophectoderm cells *via* activation of MTOR signaling in pigs [25], [43]. However, somewhat surprisingly, the present study found that exogenous EAA-induced *MTOR* mRNA expression was inhibited by combined treatment with NEAAs. This result has not been reported previously and may be explained by effects of excess amino acids on nutrient transfer capacity of embryos, which compromises the transport of certain amino acids into embryos [4]. Neutral amino acids tend to compete with each other for the same transporters [44]. Moreover, one kind of amino acid in a medium will influence content of the same and other amino acids in embryos to result in an imbalance in the overall amino acid pool within embryos which may cause phenotypic variability [45]. Because amino acids are utilized for protein biosynthesis, impaired amino acid uptake can shut down its synthesis. Leese [14] demonstrated an inverse relationship between embryo viability and metabolic activity called the “quiet embryo hypothesis.” Few amino acids have been found to disappear from the culture medium of embryos, while others appear during the cleavage stages of development [46]. We may thus assume that the inclusion of a mixture of exogenous amino acids in medium is not necessarily obligatory for preimplantation development *in vitro*; rather, the presence of all 20 free amino acids in the culture medium may have a negative impact on preimplantation embryos.

It has been suggested that use of EAA at half of Eagle's concentrations has beneficial effects on preimplantation embryo development [13]. The overall amino acid concentrations used in our study were based on the physiological data reported by a recent study [29]. On the other hand, several EAA including arginine, cystine, isoleucine, and methionine were present at apparently higher concentrations (2- to 5-fold) than estimates obtained by Iritani et al [47]. The possible explanations for this discrepancy are that these studies used different methods (excision *in vitro* and cannulation *in situ*) by which the oviduct and uterine fluids and were collected from individuals with different reproductive status (pregnancy and estrus). Also, factors such as age, breed, maternal diet, and the accuracy of diagnostic systems may contribute to increase this difference. Of the various factors considered, the data resulting from oviduct and uterine fluids in early pregnancy given by Li in [29] that may be more likely be indicative of physiological range with respect to the development of preimplantation porcine embryos than the one given by Iritani in [47]. However, there are some concerns related to the methods for collection of oviduct and uterine fluids as summarized by Leese et al. [48]. They suggested that such sampling methods are important for accurately measuring physiologically relevant concentrations of these fluids ahead of other considerations. In regard to the uncertainties in reference data, additional experiments are necessary to more precisely elucidate physiological concentrations of amino acids in tract fluids.

Amino acids are almost certainly involved in significant physiological functions at the cellular level, and they are essential in the medium employed to support a developing mammalian embryo [45]. It is also clear that specific amino acids have stimulatory and inhibitory benefits on early embryonic development *in vitro* [11]. Imprinted genes play important roles in regulating maternal supply and fetal demand for nutrients and epigenetic changes caused by environmental conditions is required for metabolic adaptation that may render them particularly prone to unfavorable environmental factors for embryonic development [49]. It should be noted that parthenogeneric embryos that are derived solely from the maternal origin, implicating that possible interaction among transcripts from the parental genomes will not be revealed in such study models. The results should be interpreted with that understanding. To date, little evidence has shed light on the mechanisms involved in the regulation of gene transcription by amino acids in preimplantation embryos. Our findings provide preliminary evidence that a mixture of exogenous amino acids influences gene expression patterns in parthenogenetic preimplantation porcine embryos. However, many questions remain unanswered. For instance, we do not know whether the observed changes resulted from stress due to inappropriate concentrations of overall amino acid levels, a subset of amino acids, or a particular amino acid in the medium. Thus, the mechanisms by which amino acids directly modulate imprinted gene expression remains unclear. Thus, more research on these issues is necessary to elucidate the association between imprinted gene expression and amino acids, which will aid the improved formulation of embryo culture media to ensure transcriptional and epigenetic stabilities with the lowest environmental impact.
